# Histologic validation of myocardial fibrosis measured by T1 mapping: a systematic review and meta-analysis

**DOI:** 10.1186/s12968-016-0313-7

**Published:** 2016-12-12

**Authors:** Kai-yue Diao, Zhi-gang Yang, Hua-yan Xu, Xi Liu, Qin Zhang, Ke Shi, Li Jiang, Lin-jun Xie, Ling-yi Wen, Ying-kun Guo

**Affiliations:** 1 Department of Radiology, Key Laboratory of Birth Defects and Related Diseases of Women and Children of Ministry of Education, West China Second University Hospital, Sichuan University, 20# Section 3 South Renmin Road, Chengdu, 610041 China; 2 Department of Radiology, State Key Laboratory of Biotherapy, West China Hospital, Sichuan University, No.37 Guoxue Xiang, Chengdu, 610041 China

**Keywords:** Cardiovascular magnetic resonance, T1 mapping, Myocardial fibrosis

## Abstract

**Background:**

Myocardial fibrosis is being increasingly recognised as a common final pathway of a wide range of diseases. Thus, the development of an accurate and convenient method to evaluate myocardial fibrosis is of major importance. Although T1 mapping is a potential alternative for myocardial biopsy, validation studies are limited to small numbers and vary regarding technical facets, and include only a restricted number of disease. A systematic review and meta-analysis was conducted to objectively and comprehensively evaluate the performance of T1 mapping on the quantification of myocardial fibrosis using cardiovascular magnetic resonance (CMR).

**Methods:**

PubMed, EMBASE and the Cochrane Library databases were searched for studies applying T1 mapping to measure myocardial fibrosis and that validated the results via histological analysis. A pooled correlation coefficient between the CMR and histology measurements was used to evaluate the performance of the T1 mapping.

**Results:**

A total of 15 studies, including 308 patients who had CMR and myocardial biopsy were included and the pooled correlation coefficient between ECV measured by T1 mapping and biopsy for the selected studies was 0.884 (95% CI: 0.854, 0.914) and was not notably heterogeneous chi-squared = 7.44; *P =* 0.489 for the Q test and I^2 = 0.00%).

**Conclusions:**

The quantitative measurement of myocardial fibrosis via T1 mapping is associated with a favourable overall correlation with the myocardial biopsy measurements. Further studies are required to determine the calibration of the T1 mapping results for the biopsy findings of different cardiomyopathies.

## Background

Myocardial fibrosis is a common histological characteristic of various heart conditions, from advanced aging to diseases such as myocardial ischemia, aortic stenosis, hypertrophic cardiomyopathy (HCM) and dilated cardiomyopathy (DCM) [1, 2]. A close relationship between myocardial fibrosis and the development of myocardial dysfunction, including impaired relaxation, precipitation of arrhythmias and impaired contractile ability [3] is associated with a wide range of diseases. Thus, the degree and distribution of the fibrosis can serve as key indicators for disease development, mortality [4], as well as prognosis [5, 6]. Therefore, the development of an accurate and convenient way to measure and assess myocardial fibrosis is of clinical significance.

**Fig. 1:**
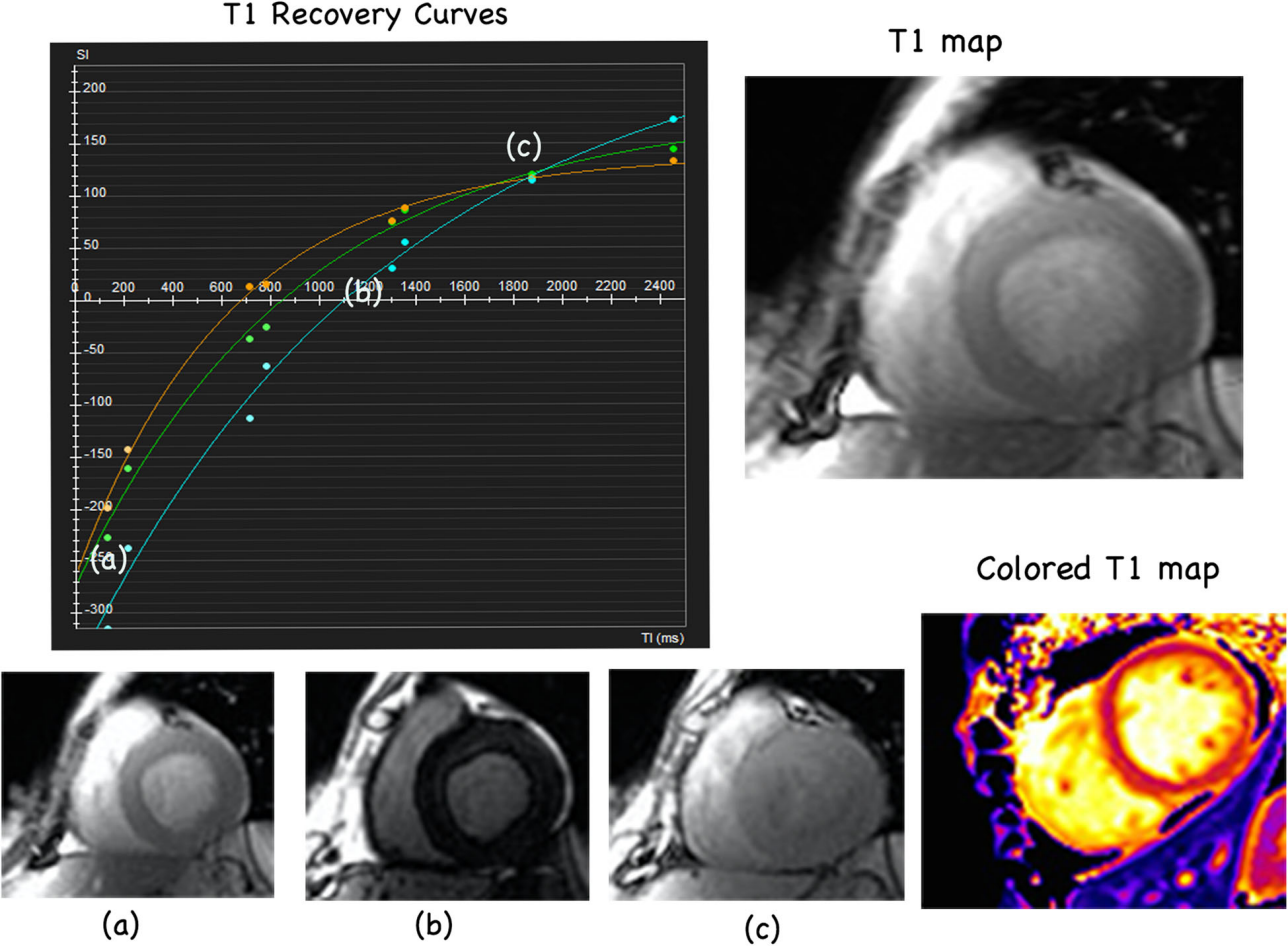
The graph on the left shows recovery curves for a septal region of interest and the blood pool, generated from images at the corresponding time point (**a**) (**b**) (**c**) in the recovery curves, shown in the bottom row, taken at different times after an inversion pulse at time = 0. The T1 for each pixel location can be used to generate a T1 map, as shown in the top-right images, and performing this technique for all pixels in the image yields a colored T1 MOLLI map (*bottom right*)

Traditionally, endomyocardial biopsy (EMB) was the only method by which to visualise and measure the collagen volume fraction (CVF) to determine the degree of fibrosis. Nevertheless, the clinical application of EMB is largely limited by its invasiveness, requirements for skilled operators, the patient factors [7], and sampling bias. Recently, the establishment of T1 mapping Cardiovascular Magnetic Resonance (CMR) [8], provides a novel and non-invasive method of visualising and quantifying myocardial fibrosis (Fig. 1). This quantification includes native or pre-contrast T1, post-contrast T1 and the expansion of the myocardial extracellular volume (ECV). Pre-contrast T1 and post-T1 are used before and after gadolinium injection respectively, and ECV measures the extracellular volume after gadolinium injection [9]. To date, three main IR-prepared CMR sequences are used in T1 mapping including standard Look-Locker(LL) sequence, modified Look-Locker MOLLI sequence and shortened-MOLLI (ShMOLLI) sequence, according to the previous reviews over techniques [10]. Several studies have been performed to examine the correlation between the T1 mapping results and the collagen measured via biopsies; the majority of the validation studies show favorable correlation. However, these studies are limited by small subject numbers and vary regarding the technical details, as well as the disease types included. A previous review on T1 mapping also mentioned the need to examine and compare the three frequently used means of interpreting T1 mapping results to provide a more systematic and credible evaluation of the performance of T1 mapping for the assessment of myocardial fibrosis [11]. To date, there have been no quantitative systematic reviews summarising the validation tests. Therefore, we conducted a systematic review and meta-analysis to objectively evaluate the performance of T1 mapping for quantitative measurement of myocardial fibrosis using CMR. We also summarised the variations in the technical details used in these studies to provide possible avenues for further research in this field.

## Methods

### Search strategy

Computerised bibliographic search of the online databases was performed using PubMed, EMBASE and the Cochrane Library. We also performed a hand-search for publications through the references listed in the selected studies. Moreover, we also searched within specific journals related to cardiac imaging, including *Radiology, Circulation, JACC, Journal of Cardiovascular Magnetic Resonance* and the *European Heart Journal*. No limits were set regarding language and any foreign papers were translated.

We confined the publication date to between 2005 and 2015. Our searches were performed using variations or combinations of the following keywords: ‘magnetic resonance imaging’; ‘Cardiovascular Magnetic Resonance (CMR)’; ‘T1 mapping’; ‘myocardial extracellular volume (ECV)’; ‘myocardial fibrosis’; ‘diffuse fibrosis’; ‘validation’; ‘endomyocardial biopsy (EMB)’ and ‘collagen volume fraction (CVF)’.

### Study selection

The eligibility of studies was independently assessed by two reviewers in an unblinded and standardised manner. Any disagreement was resolved by discussion and referring the study to a third reviewer. Based on the Preferred Reporting Items for Systematic Reviews and Meta-analysis (PRISMA) guidelines, the selection of studies was based on the type of disease, the methods used to perform T1 mapping, the procedures used for the biopsy and histochemical analyses of the CVF, and the appropriateness of the statistical methods used to test and present the correlation results. Since it remains an issue of debate as to which sequence is the best for performing T1 mapping and previous studies did not show significant differences in their ability to quantify fibrosis via different scan sequences [12–15], we did not exclude any studies due to the use of different scan sequences, provided that the T1 mapping for analysis was aquired appropriately.

Studies were selected for further analysis if they met the following criteria: 1) indicated the specific disease types and number of patients recruited in the study; 2) applying either appropriate technology (i.e. EQ-CMR, MOLLI, ShMOLLI, FLASH and Look-Locker) to acquire T1 mapping images and using one or a combination of the native T1 time, post-contrast T1 time and ECV to present the results, and using a magnetic field ≥1.5 T; 3) validating the quantitative imaging results with CVF measured from a biopsy and 4) appropriate statistical methods applied to calculate the validation results and interpret the results in either r or r^2^.

Studies were excluded if they: 1) did not perform quantitative validation tests or provide specific correlation coefficients; 2) did not apply T1 mapping-related imaging methods to acquire the ECV and 3) were studies using in vitro or animal models. Finally, if two or more studies reported the same experiment, we selected the one that was the most informative.

### Quality assessment

The study design, patients recruited and statistical methods applied in each study were considered primarily for the quality assessment of the study by referring to the items in The Quality Assessment of Diagnostic Studies (QUADAS). QUADAS is a tool developed in 2003 [16, 17] and renewed in 2011 [18] that is used to assess the quality of diagnostic studies. Fourteen items were considered with this tool to rate the index and reference standard tests.

### Data extraction

To best extract the data from studies and avoid bias, we prepared a data extraction sheet based on the data extraction template provided by Cochrane Consumers and the Communication Review Group. Two authors simultaneously extracted the data and checked the extracted data for each other. Any disagreements were settled by referring the data to a third author and were decided by a discussion between the three individuals. We collected and recorded the following details for data analysis: record number, author, article title, the year of publication, publication (journal), study design, inclusion/exclusion criteria, imaging methods (e.g. sequence and magnetic field), the number of patients included, statistical analysis methods and outcomes (Pearson or Spearman correlation coefficient (r) and 95% confidence intervals (CIs) were calculated). For the correlation coefficients, we recorded the |r| between the histology results and the three main T1 mapping measurements, including the ECV, post-contrast T1 time and native T1 time.

To calculate the CIs for each correlation coefficient, we performed a Fisher’s r-to-z transformation of the Pearson coefficients to convert each correlation coefficient into an approximately normal distribution and then acquired the upper and lower confidence intervals accordingly [19].

Define:

$$ z=0.5\times \ln \Big(\frac{1+\left|\mathrm{r}\right|}{1-\left|\mathrm{r}\right|\Big)}\;\mathrm{z}\mathrm{l}=\mathrm{z}-\frac{1.96}{\sqrt{\mathrm{N}-3}}\;\mathrm{z}\mathrm{u}=\mathrm{z}+\frac{1.96}{\sqrt{\mathrm{N}-3}} $$

The confidence interval of |r|: $\left(\frac{\ {\mathrm{e}}^{2\mathrm{z}\mathrm{l}}-1}{{\mathrm{e}}^{2\mathrm{z}\mathrm{l}}+1}\mathrm{t}\mathrm{o}\frac{{\mathrm{e}}^{2\mathrm{z}\mathrm{u}}-1}{{\mathrm{e}}^{2\mathrm{z}\mathrm{u}}+1}\right)$

To qualitatively summarise the results of different studies, we recorded and compared the technical differences for T1 mapping and any variations of the correlation results while T1 mapping was used in a different group of patients to further discuss the characteristics.

### Data synthesis

Our analysis included the summary estimates of the correlation coefficients to assess the overall correlation between the CMR measurements with the histology results. Thus, for the pooled estimates of the correlation coefficients, we computed the summary statistics across the individual studies. These were then combined to produce the pooled estimates of the correlation coefficients and confidence intervals for the comparison, which were weighted according to the number of patients in the sample. The meta-analysis was performed using the fixed effect or random model to combine and calculate the cumulative effects of the correlation coefficients according to the heterogeneity of the data; this was assessed using both the Cochran Q test and the I^2^ statistic, based on the percentage of the total variation among the studies instead of chance. *P <* 0.05 for the Q test or I^2^ statistic >50% indicated the presence of significant heterogeneity across the selected studies and a random-effect model was then applied to calculate the tau value. For the limited number of the studies that were finally included, the quality of the correlation could not simply be classified according to previous studies. We did not perform an analysis of publication bias and we used Stata/SE 12 to perform the meta-analysis.

### Search strategy and study selection

The online search initially yielded 868 literature citations. In total, 628 studies were excluded after reviewing the titles and keywords due to a lack of relevance. One author reviewed 240 abstracts and selected 29 studies for the full-text evaluation. Further reading excluded additional papers according to the criteria defined above. Seven studies were ruled out for not using CVF or contrast-enhanced T1 mapping as an assessment method. Five studies were excluded due to the lack of reported correlation coefficients and two were excluded due to the use of animal models or post-mortem analyses.

**Fig. 2:**
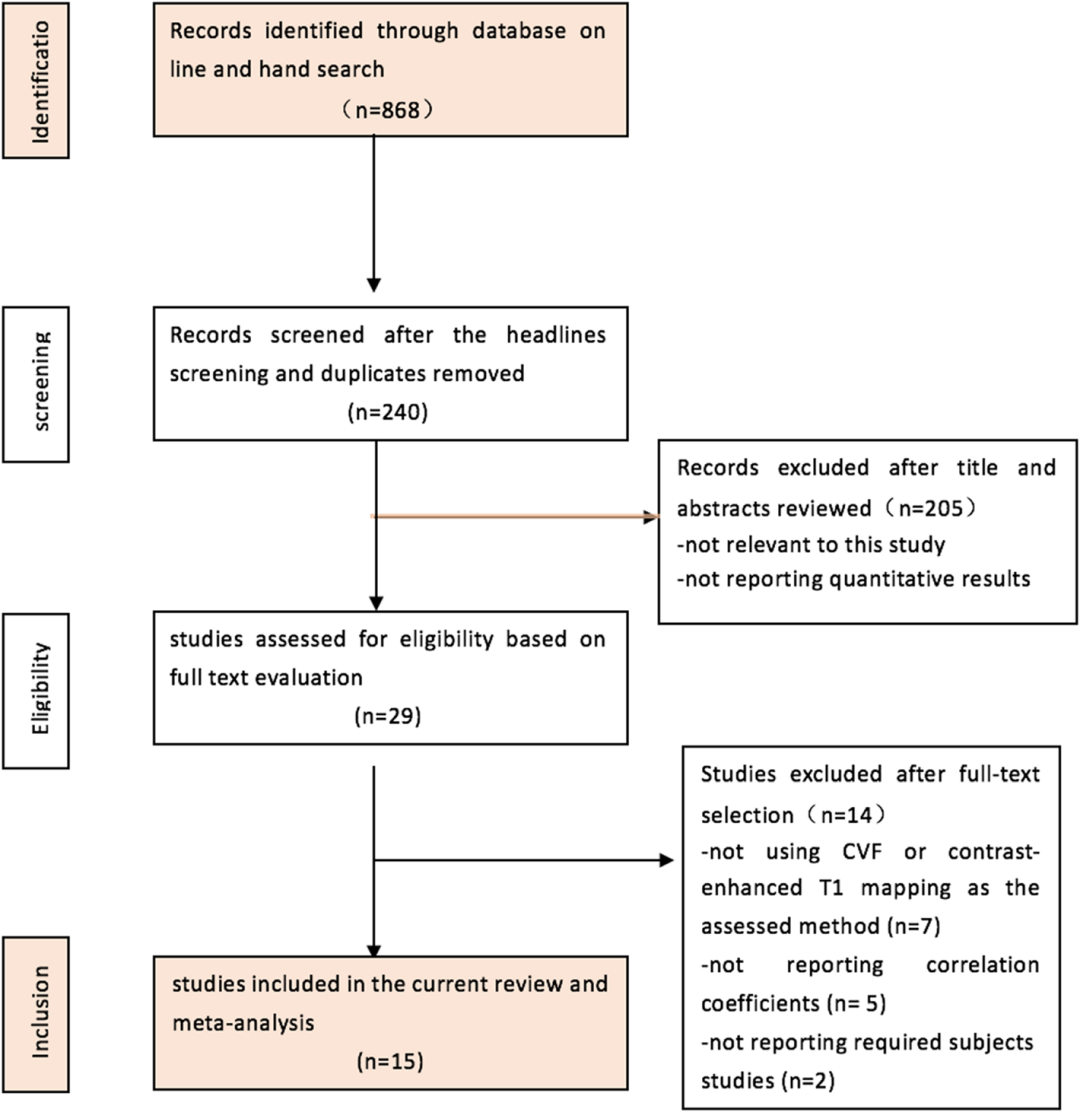
Flow diagram of the study selection process

Finally, 15 studies published from 2010 to 2015 fulfilled our inclusion criteria and 23 experimental results were analysed because some of the studies included both ECV and pre- or post-contrast T1 relaxation time as the CMR measurements. The PRISMA flow chart depicting the process of the systematic literature search and study selection is illustrated in Fig. 2. Five studies were from conference articles.

## Results

### Study design characteristics

The detailed information for the 15 selected studies is presented in Table 1. The total number of patients recruited in the studies was 308. The patients were diagnosed with a wide range of heart diseases, including valvular heart disease (113), cardiomyopathy (113) and heart failure or dysfunction caused by other reasons (e.g. cardiac amyloidosis) (82). Three studies applied the ShMOLLI sequence followed by the process described by Piechnik. Another 3 studies applied FLASH and 7 studies applied MOLLI for T1 mapping. Moreover, one study applied equilibrium contrast and one study used Look-Locker for T1 mapping. All of the selected experiments provided credible data. In four studies, the correlation coefficient was expressed as r^2^ and another study used Kendall’s test to calculate the correlation coefficient.

**Table 1:** included studies and patients’ characteristics. AS: aortic stenosis; HCM: hypertrophic cardiomyopathy; DCM: dilated cardiomyopathy; MR: mitral regurgitation; ARVD: arrhythmogenic right ventricular dysplasia; HFpEF: heart failure with preserved ejection fraction; HFrEF: heart failure with reduced ejection fraction; ICM: ischemic cardiomyopathy

Year	Author	Journal	Number of patients	Field	Disease type	Scan
2010	F.A.S [28]	circulation	26	1.5 T	AS(18) HCM (8)	EQ-CMR
2012	WSK [27]	JCMR	12	1.5 T	AS (12)	shMOLLI
2012	FM [26]	JCMR	18	1.5 T	AS(18)	shMOLLI
2013	WSK [24]	JACC	18	1.5 T	AS (18)	shMOLLI
2013	MCA [22]	Circulation	6	1.5 T	DCM(3) ischemia (3);	MOLLI
2014	ADSP [37]	JCMR	24	1.5 T	DCM(24)	MOLLI
2015	GY [20]	JCMR	20	3 T	DCM (20)	MOLLI
2015	DMDRC [21]	JCMR	31	3 T	SAS (12),severe aortic regurgitation (9),MR (10)	MOLLI
2015	KA [35]	JCMR	36	1.5 T	heart failure (HFpEF (22), cardiac amyloidosis (7), HFrEF (3), MR (4))	MOLLI
2015	LG [38]	JCMR	4	1.5 T	AS (4)	MOLLI
2016	KA [39]	JACC	36	1.5 T	heart failure (28), valvular heart disease (8)	MOLLI
2013	MJ [23]	Circulation	9	1.5 T	HFpEF (9)	FLASH
2008	IL [25]	JACC	9	1.5 T	heart transplantation recipients (9)	FLASH
2015	IL [40]	EHJ	12	1.5 T	HCM(8), DCM (2) ICM(1),restrictive(1)	FLASH
2012	SCT [41]	Radiology	47	1.5 T	DCM(13), myocarditis (11), infiltrative /restrictive cardiomyopathy(22),suspected ARVD (1)	Look-Locker

All of the studies excluded patients who had contraindications to CMR or the contrast agent, as well as patient data after considering the imaging quality. Among the studies reporting exclusion details: four studies [20–23] reported excluding patients with significant coronary artery disease or myocardium infarction that was too severe and another three studies [24, 25] reported excluding patients with severe arrhythmia. Two studies [26, 27] were reported to exclude patients based on their inability to hold their breath during CMR. Furthermore, one study [28] also excluded patients with other organ dysfunction, such as renal failure.

**Fig. 3:**
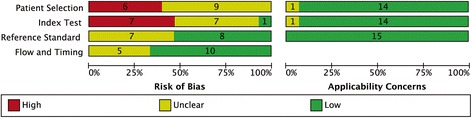
Study quality evaluated by QUADAS-2 tool. Grouped bar chart displays the cumulative score of the 15 included studies for each fields of the QUADAS questions. Green bar = “low” risk, yellow bar = “unclear” risk, and red bar = “high” risk. The questions are listed in Table 2

The quality assessment results are presented in Fig. 3 and the items are shown in Table 2. Although all studies provided detailed information pertaining to the type of disease included, the CMR findings, as well as the technology they used to acquire the T1 mapping, the majority of studies did not clearly state the time interval between the CMR and biopsy measurements. There were 12 out of 15 studies that clearly indicated the analysis of the CMR and biopsy results were blinded.

**Table 2:** QUADAS-2 questions. Results of the analysis for the quality evaluation are shown in Fig. 3

QUADAS-2 Question
A. patient selection
Was a consecutive or random sample of patient enrolled?
Was a case control design avoided?
Did the study avoid in appropriate exclusion?
B. index test
were the index test interpreted without knowledge of results of the reference standard?
if a threshold was used, was pre- specified?
C. Reference standard
Is the reference standard likely results interpreted without knowledge of the result of the index tests?
D. flow and timing
Was there an appropriate interval between index tests and reference standard?
Did all the patients receive the same reference standard?
Were all patients included in the analysis?

### Quantitative review

Following the Fisher’s r-to-z transformation method, we obtained the CIs for 11 out of 15 studies since this transformation is preferred when the number of patients is greater than ten. The results are presented in Table 3. For the 11 studies we chose, nine studies reported ECV, five reported the post-contrast T1 time and three studies reported the native T1 time as the quantitative T1 mapping results.

**Table 3:** Correlations coefficient between three T1 mapping results with histological CVF

Study	Number of patients	Correlation coefficient	
		|r|	CI
Native T1 time			
MCA, 2013	6	0.95	-
GY, 2010	20	0.67	[0.32, 0.86]
DMDRC, 2012	31	0.15	[–0.22,0.48]
KA, 2015	36	0.66	[0.42, 0.81]
Post-T1 time			
MJ,2013	9	0.98	-
IL, 2008	9	0.7	-
IL, 2015	12	0.78	[0.37, 0.94]
SCT, 2012	47	0.57	[0.37, 0.74]
WSK, 2013	18	0.51	[0.06, 0.78]
MCA, 2013	6	0.74	-
DMDRC, 2015	31	0.36	[0.01, 0.63]
KA, 2015	36	0.68	[0.45, 0.82]
ECV			
F.A.S, 2010	26	0.89	[0.88,0.96]
WSK, 2012	12	0.75	[0.31,0.93]
FM, 2012	18	0.83	[0.59,0.93]
WSK, 2013	18	0.84	[0.61,0.94]
MCA, 2013	6	0.95	-
ADSP, 2014	24	0.85	[0.68,0.93]
GY, 2015	20	0.71	[0.38,0.88]
DMDRC, 2015	31	0.78	[0.59,0.89]
KA, 2015	36	0.91	[0.83,0.95]
LG,2015	4	0.91	-
KA,2016	36	0.49	[0.19,0.71]

**Fig. 4:**
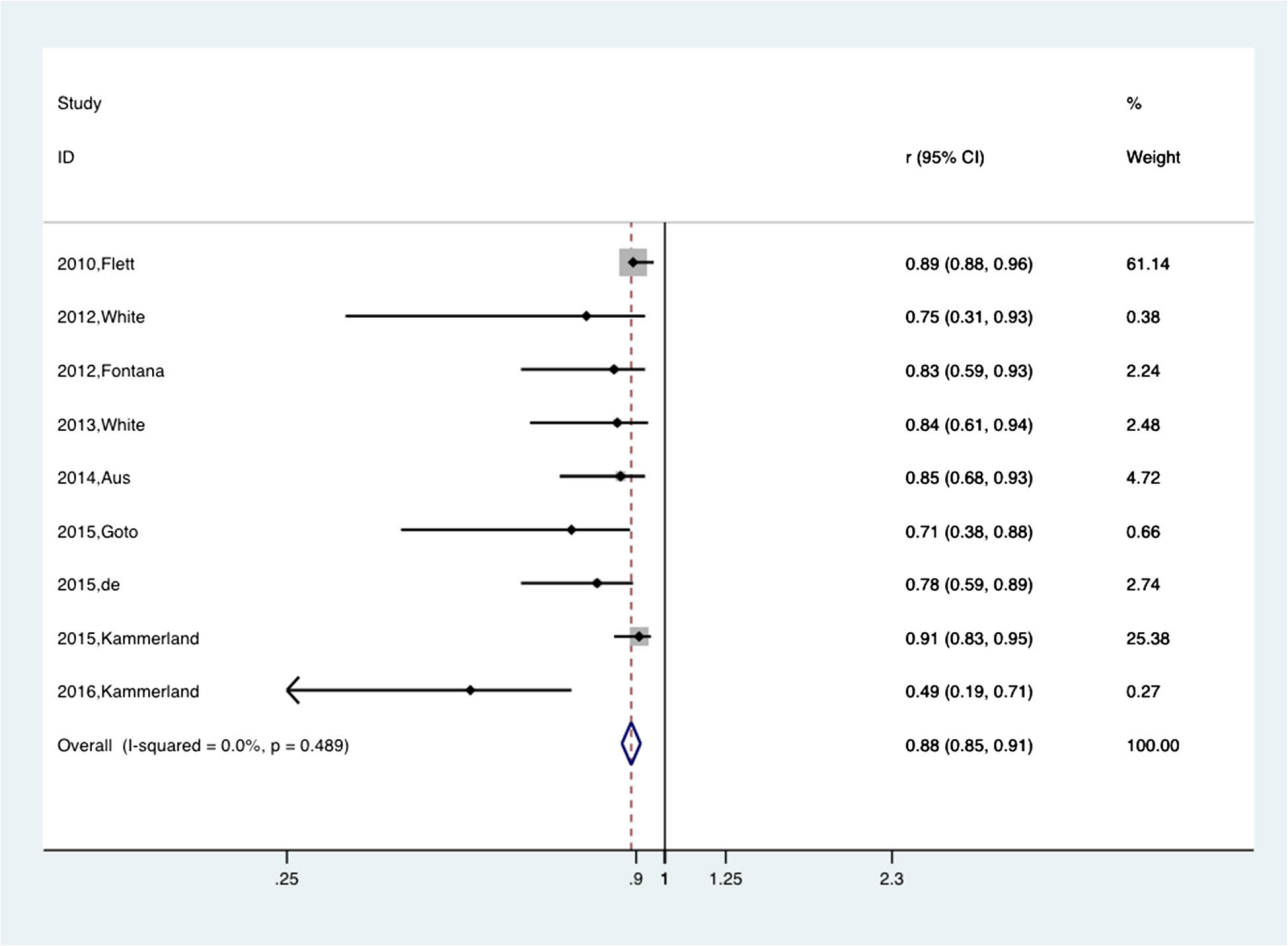
Forest plot of the correlation coefficients between ECV and histology CVF measurement

**Fig. 5:**
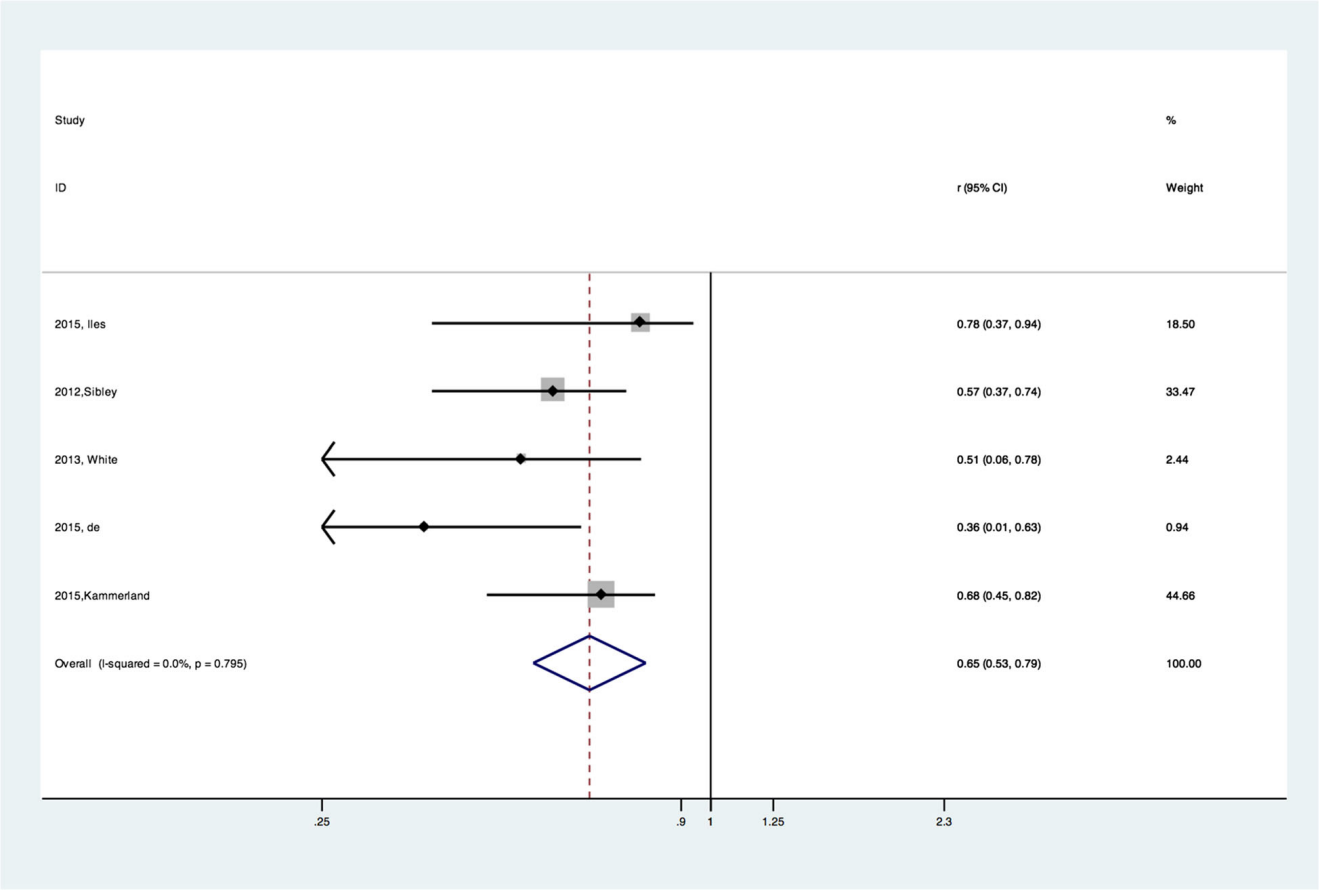
Forest plot of the correlation coefficients between post-contrast T1 time and histology CVF measurement

**Fig. 6:**
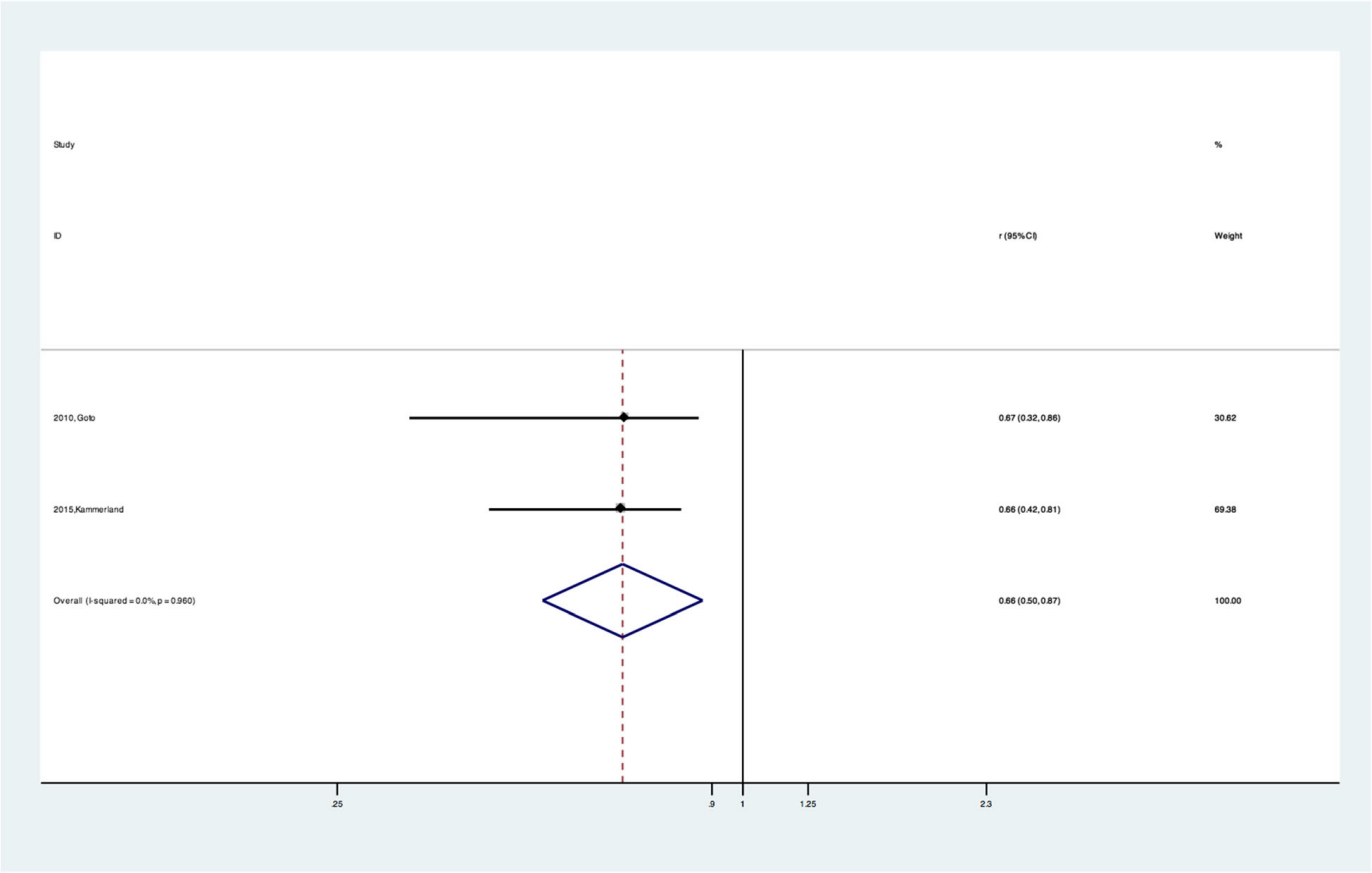
Forest plot of the correlation coefficients between native T1 time and histology CVF measurement

The pooled r for the nine studies describing the overall estimated correlations between CVF and ECV was 0.88 (95% CI:0.854, 0.914) and was not notably heterogeneous (chi-squared = 7.44, *P =* 0.489 for the Q test and I^2^ = 0.00%) (Fig. 4). On the other hand, the pooled |r| for the other five studies applying post-contrast T1 by merging the correlations between CVF and the post-contrast T1 time was 0.65 (95% CI: 0.53, 0.79) and was also not notably heterogeneous (chi-squared = 1.68, *P =* 0.795 for the Q test and I^2^ = 0.00%) (Fig. 5). For the native T1 time, only two studies provided available data. The calculated pooled |r| was 0.66 (95% CI: 0.50, 0.87) and was not notably heterogeneous (chi-squared = 0.00, *P =* 0.96 for the Q test and I^2^ = 0.00%) (Fig. 6).

### Qualitative review

Among the 15 studies that were initially selected, two used 3 T while the others used 1.5 T MR. No major differences were noted. In consideration of the scanning techniques, three studies used the ShMOLLI sequence when performing the T1 mapping measurements and reported the advantages of choosing this method [24, 26, 27]. Fontana’s [26] experiments compared the multibreath-hold techniques (e.g. fast low angle single shot inversion recovery; ‘multibreath-hold FLASH-IR’) and ShMOLLI. Moreover, according to their study, compared to the FLASH-IR technique, the ShMOLLI method was easier to perform successfully on patients and had superior reproducibility and a higher correlation with the histology CVF% [25].

Similar to the method used to achieve equilibrium distribution in patients, White’s study [24] compared the differences between the use of a primed contrast infusion (equilibrium contrast-CMR[EQ-CMR]) and the dynamic equilibration achieved by delayed post-bolus measurements. The results of this study exhibited a favourable correlation between the ECV measured by the dynamic equilibration with the CVF acquired from the biopsy sample (r^2^ = 0.69). Regardless of the fact that this correlation was not as high as the EQ-CMR method (r^2^ = 0.71), the two methods did not exhibit a significant difference. The author also suggested the use of the EQ-CMR method if the ECV was found to be over 0.4.

Concerning the T1 time measurements, one study by Miller [22] employed the MOLLI bolus method and found that a 15-min post-contrast T1 time was associated with a better correlation degree between the ECV and CVF than the 10-min post-contrast T1 time.

## Discussion

Our meta-analysis and systematic review demonstrates a favorable overall correlation between the CMR measurements and the histological measurements from the biopsy. An accurate calculation of the pre- and post- T1 relaxation time, as well as ECV via T1 mapping can reflect the degree of myocardial fibrosis in patients with a wide range of diseases.

This is the first study to quantitatively combine the previous histological validation tests using T1 mapping technology for the measurement of myocardial fibrosis. The studies we included were modestly selected following the PRISMA guidelines. Quality assessment through QUADAS reveals that the studies from which we extracted data, performed credible and high-quality validation experiments. The heterogeneity test revealed no heterogeneity among the studies we selected.

Myocardial fibrosis is the final pathway in many disease states and it can be insidious since only enough amount of fibrosis or accumulation can induce prominent ventricular incompliance and heart dysfunction. Although being considered as the gold standard, EMB examination is not routinely recommended. Non-invasive quantification assessments include serum collagen biomarkers (eg. carboxy-terminal pro-peptide of pro-collagen type I (PICP) and ratio of matrix metalloproteinase type 1 to tissue inhibitor of metalloproteinase type 1 (MMP-1/TIMP)) and imaging techniques [29]. Previous study reported a moderate correlation between PICP and CVF (*r =* 0.471) in hypertensive patients [30] and according to the recent reviews, CMR techniques including T1 mapping and Late Gadolinium Enhancement (LGE) have superior specificity than the commonly used serum biomarkers [31]. In fact, LGE CMR has excellent performance for the diagnosis of some patients with myocardial fibrosis and has been recognised as a replacement approach for the diagnosis of patients with focal myocardial fibrosis using biopsies [32, 33]; however, this technology remains limited by its requirement for normal tissues as reference samples and thus, cannot provide an accurate evaluation of diffuse myocardial fibrosis [34].

Based on an imaging mechanism similar to LGE, T1 mapping is used to map out the image-based signal intensities according to myocardium’s longitudinal relaxation time. The differences in signal intensity can be directly interpreted as variations in the amount of fibrosis in the scanned heart tissue. Therefore, it is not necessary to use normal tissues as a standard reference to interpret the results and both focal and diffuse fibrosis can be quantified. According to our results, all three of the T1 mapping measurements can have a favourable correlation with the histological results, which can serve as the basis for studies that further test the prognostic value of the T1 mapping results. Among these three methods, ECV has the highest correlation value (*r =* 0.88) while native T1 has the lowest (*r =* 0.66). Theoretically, with the use of a contrast injection, the tissue heterogeneity should be easier to detect since the discrepancies in the washout time between the fibrotic and normal tissues add to the gap of the T1 time, as gadolinium can potently shorten the T1. However, post-T1 can only partly reflect the fibrosis since gadolinium does not cross cell membranes and factors such as renal excretion rate would also have a role. Thus ECV, after taking the blood gadolinium as a reference, is supposed to better reflect the collagen content, as well as the fibrosis degree. Furthermore, a limited number of studies were used for the pooled r calculation, in which the native T1 results exhibited relatively high variability between the different studies. Meester de Ravenstein et al [21] reported that neither the amount of LGE nor the native pre-contrast myocardial T1 time were associated with significant correlation results for the histology CVF. In contrast, in Yoshitaka Goto’s [20] study, the native T1 time exhibited a better correlation with the histology (*r =* 0.673).

We noted that diffuse myocardial fibrosis might be better than focal fibrosis for quantification using the T1 mapping technique. Flett et al [28] reported that the observed correlation was higher in myocardial fibrosis with aortic stenosis than in those with increased focal cardiomyopathy. One potential reason for this high correlation with aortic disease might be that the vasodilation and microcirculation conditions caused by various vascular abnormalities adds to the accumulation of extracellular collagen. However, since these substances also contribute to disease progression and the heart load, the T1 mapping results may still be a better interpretation under these circumstances rather than techniques that only take fibrosis into consideration. According to the study by Andreas et al, the correlation was substantially greater when they included patients with a diagnosis of cardiac amyloidosis [35]. In addition, a previous study [9] assumed that the first clinical application of T1 mapping might be to examine myocardial fibrosis in rare diseases and once the diagnosis and prognostic value of this method is agreed upon, it will aid in the intervention of more common cardiovascular diseases. However, considering that myocardial fibrosis is a shared pathological characteristic, the presence of good stable correlations and low heterogeneity among the studies we analysed provides support for T1 mapping.

### Study limitations

The major limitation of our study is the limited number of reports that we included in our meta-analysis. Moreover, due to the rapid development and optimisation of the T1 mapping sequence, we did not identify a sufficient number of studies for each sequence to conduct a subtype analysis for T1 mapping according to both the scanning sequence and the type of diseases that cause myocardial fibrosis. However, the value of this meta-analysis is that we attempted to indicate (regardless of the scanning protocol and aetiology of myocardial fibrosis) whether quantitative T1 mapping can provide a useful reference to assess the degree of myocardial fibrosis.

The pooled r for native T1 is another concern of our study. Although the pooled r showed a good performance of native T1 for predicting myocardial fibrosis, the limited number of studies, as well as the poor quality made this result less credible. More studies with larger number of patients and multi-centre study are still needed to better clarify this issue.

We also included used both 1.5 T and 3 T magnetic fields to perform T1 mapping. However, there appears to be some dispute on whether 1.5 T or 3 T will have an effect on the post-contrast T1 mapping measurements [36]. Therefore, we did not exclude the two studies that applied 3 T CMR for T1 mapping.

In addition, a good correlation between the two methods is not enough to completely replace the EMB method. Thus, additional studies are still required to determine an optimal and accurate transformation function from the ECV value or T1 relaxation time to CVF. Using such information, the clinical application of T1 mapping can be further propagated.

## Conclusion

The CMR measurements of myocardial fibrosis through T1 mapping correlates well with the CVF measured through EMB, especially for patients with diffuse myocardial fibrosis. Further studies are required to test and acquire the detailed transformation function from the ECV or T1 time to CVF based on different degrees of myocardial fibrosis or the type of cardiomyopathy.

## Abbreviations

CMR: Cardiovascular magnetic resonance
CVF: Collagen volume fraction
DCM: Dilated cardiomyopathy
ECV: Extracellular volume
EMB: Endomyocardial biopsy
EQ-CMR: Equilibrium contrast-CMR
HCM: Hypertrophic cardiomyopathy
LGE: Late Gadolinium Enhancement
QUADAS: Quality Assessment of Diagnostic Studies
